# Nowhere to Go but Up: Impacts of Climate Change on Demographics of a Short-Range Endemic (*Crotalus willardi obscurus*) in the Sky-Islands of Southwestern North America

**DOI:** 10.1371/journal.pone.0131067

**Published:** 2015-06-26

**Authors:** Mark A. Davis, Marlis R. Douglas, Colleen T. Webb, Michael L. Collyer, Andrew T. Holycross, Charles W. Painter, Larry K. Kamees, Michael E. Douglas

**Affiliations:** 1 Department of Biology, Colorado State University, Ft. Collins, Colorado, United States of America; 2 Department of Biological Sciences, University of Arkansas, Fayetteville, Arkansas, United States of America; 3 Department of Biology, Western Kentucky University, Bowling Green, Kentucky, United States of America; 4 School of Life Sciences, Arizona State University, Tempe, Arizona, United States of America; 5 New Mexico Department of Game and Fish, Santa Fe, New Mexico, United States of America; 6 Department of Biology, University of Texas-El Paso, El Paso, Texas, United States of America; State Natural History Museum, GERMANY

## Abstract

Biodiversity elements with narrow niches and restricted distributions (i.e., ‘short range endemics,’ SREs) are particularly vulnerable to climate change. The New Mexico Ridge-nosed Rattlesnake (*Crotalus willardi obscurus*, CWO), an SRE listed under the U.S. Endangered Species Act within three sky islands of southwestern North America, is constrained at low elevation by drought and at high elevation by wildfire. We combined long-term recapture and molecular data with demographic and niche modeling to gauge its climate-driven status, distribution, and projected longevity. The largest population (Animas) is numerically constricted (N = 151), with few breeding adults (N_b_ = 24) and an elevated inbreeding coefficient (ΔF = 0.77; 100 years). Mean home range (0.07km^2^) is significantly smaller compared to other North American rattlesnakes, and movements are within, not among sky islands. Demographic values, when gauged against those displayed by other endangered/Red-Listed reptiles [e.g., Loggerhead Sea Turtle **(** *Caretta caretta* **)]**, are either comparable or markedly lower. Survival rate differs significantly between genders (female<male) and life history stages (juvenile<adult) while a steadily declining population trajectory (r = -0.20±0.03) underscores the shallow predicted-time-to-extinction (17.09±2.05 years). Core habitat is receding upwards in elevation and will shift 750km NW under conservative climate estimates. While survival is significantly impacted by wildfire at upper elevations, the extinction vortex is driven by small population demographics, a situation comparable to that of the European Adder (*Vipera berus*), a conservation icon in southern Sweden. Genetic rescue, a management approach successfully employed in similar situations, is ill advised in this situation due to climate-driven habitat change in the sky islands. CWO is a rare organism in a unique environment, with a conserved niche and a predisposition towards extinction. It is a bellwether for the eventual climate-driven collapse of the Madrean pine-oak ecosystem, one of Earth’s three recognized megadiversity centers.

## Introduction

Earth’s climate is increasingly variable [1], as gauged across a variety of unique habitats. Climate-related metrics derived for southwestern North America present a confusing mix [2]. In many cases they are either far above average (i.e., diurnal temperatures; premature snowmelt) or far below (i.e., precipitation amounts, snowpack levels, reservoir capacities). This variability has been broadened and extended by other climatological aspects as well. Loss of extremely cold winter temperatures, for example, promotes survival of larval Pine Beetle (*Dendrocnotus* sp.), and with cascading effects. Those more proximate relate to greater infestation rates that, in turn, translate into amplified tree mortalities and fire intensities, whereas those more distal promote bivoltine life histories [3] and the depletion of regional carbon sinks. The end result is that ~18% of southwestern forests have now been lost, a value that will exceed 50% when two droughts similar to the most recent are recorded [4].

Climate-driven forest depletions also impact biodiversity. Here, range shifts are of particular concern, yet are often perceived as being transitory, particularly amongst generalist species with broad tolerances and plastic responses [5]. Vulnerabilities of specialists, on the other hand, are often overlooked, due largely to their conserved ecologies, restricted environmental ranges, and limited trophic breadths [6]. Species most susceptible to habitat-induced range-shifts are specialists with distributions easily contained within a 100x100 km grid (i.e., <10,000 km^2^) and are thus deemed ‘short-range endemics’ (SREs) [7]. While climate change is most often recognized at continental or global levels, its impacts are most severe on species that are localized and relatively constrained, and whose demise often cues the disassembly of community structure [8].

**Fig 1:**
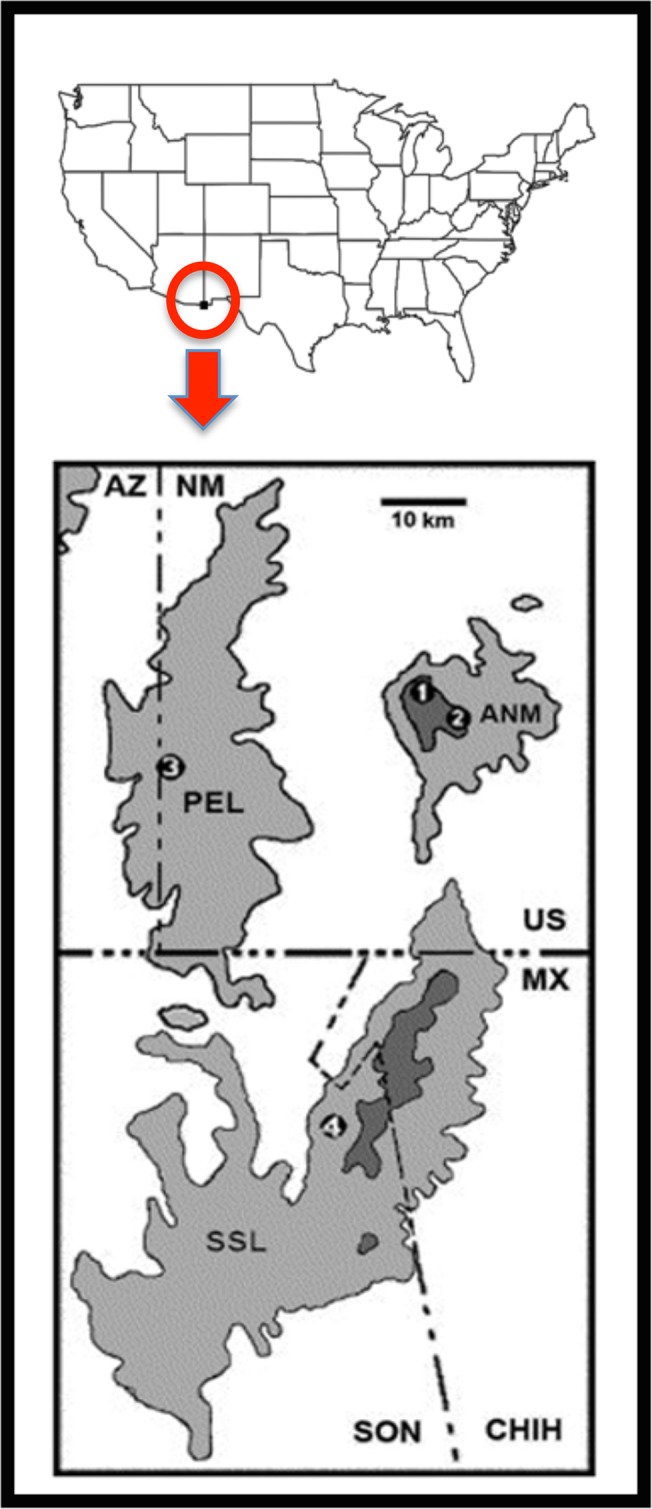
Distribution of Crotalus willardi obscurus in North American (top picture), and magnified into a perspective of the three sky-islands in southwestern North America (bottom picture) that straddle southeastern Arizona (AZ), southwestern New Mexio (NM), and north-central México (MX). PEL = Peloncillos Mountains (AZ); ANM = Animas Mountains (NM); SSL = Sierra San Luis Mountains (México). Numbers indicate specific locations of study sites, two of which are within Animas Mountains: (1) = West Fork Canyon (ANM-W); (2) = Indian Creek (ANM-I); (3) = Peloncillos Mountain; (4) = Sierra San Luis Mountain.

Short Range Endemics are often found within biodiversity ‘hotspots’ (i.e., exceptional concentrations of endemics within receding habitats; [9]), and many of those constituent SREs are afforded some level of protection. Long-term persistence of SREs is particularly perilous in montane environments where a shifting climate has manifold effects. For example, available habitat in these regions inexorably shifts to higher elevations as climate warms [10], whereas the remnant and remaining habitat is immediately susceptible to instantaneous wildfires [11]. This analogy aptly describes our short-range endemic study species, the federally threatened New Mexico Ridge-nosed Rattlesnake, *Crotalus willardi obscurus* (= CWO, Fig 1) and its restricted and specialized habitat within the elevated Madrean Pine-Oak ecosystem of southwestern North America. Here, conservation issues have rapidly escalated from those specific and taxon-centric towards a larger environmental concern, the collapse of the Madrean woodlands, one of three global “megadiversity” centers [12].

Environmental, demographic, and genetic aspects act synergistically to drive extinction vortices [13, 14], and herein we employed contemporary approaches to evaluate each of these with regard to our SRE. In so doing, we expanded earlier research [15] by estimating the following parameters for our sky island populations: 1) survivorship, fecundity, mortality, and population size by gender and ontogeny; 2) home range and movement; 3) survivability following stochastic wildfire; 4) intrinsic demographic aspects as reflected in population genetic parameters; and 5) both current and future climate envelopes. To place our demographic results within a global context, we also reported values for other North American rattlesnakes, as well as two other listed reptiles for which data are available (i.e., Loggerhead Sea Turtle, *Caretta caretta*; European Adder, *Vipera berus*, in southern Sweden). The recovery of the former species is seriously impeded by anthropogenic activities that impact nesting and foraging habitats, as herein [16, 17], whereas the latter provides a widely known and taxonomically congruent benchmark [18] against which our study species can be contrasted. This is particularly appropriate given well-documented attempts at genetic rescue for the Adder [19, 20] and its subsequent relapse into population decline [21].

## Materials and Methods

### *Crotalus willardi obscurus* as a Sky Island Short Range Endemic

The Pleistocene fragmented the southern Rocky Mountains and northern Sierra Madre Occidental creating ‘sky islands’ [22]–areas with vertically segregated life zones within Sonoran and Chihuahuan deserts [23]. Each sustains an endemic and characteristic community straddling two floristic (Neotropic/Holarctic) and two faunal (Neotropic/Nearctic) realms [12], all under pressure from concomitant and conflicting anthropogenic demands and expectations. Scientists and resource agencies work to conserve biological diversity whereas others seek commercial and/or consumptive access.

**Fig 2:**
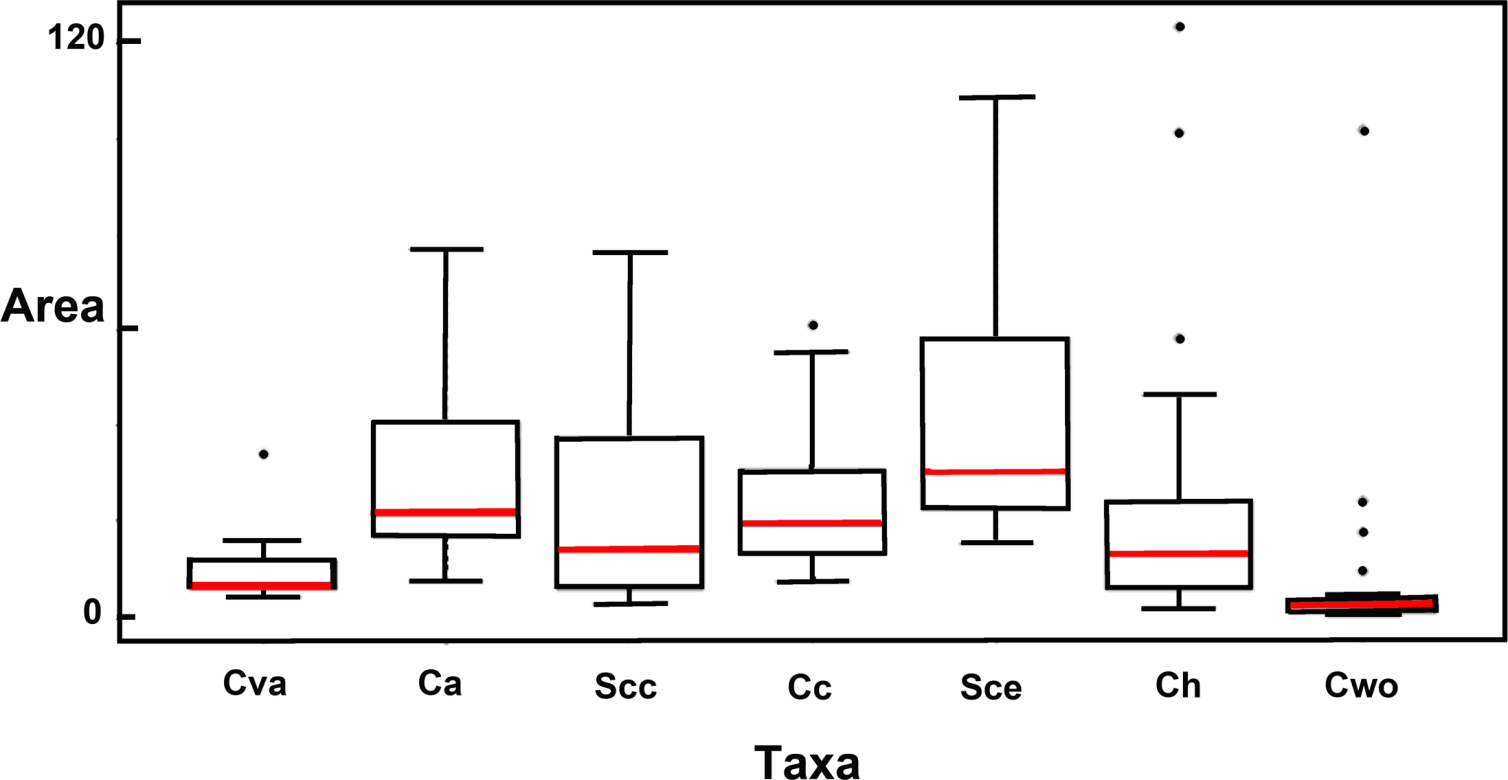
Mean home range estimates for Crotalus willardi obscurus and six other rattlesnake species for which similar data were available. Horizontal red bar = mean value. Cva = *Crotalus viridis abyssus*; Ca = *Crotalus adamanteus*; Scc = *Sistrurus catenatus catenatus*; Cc = *Crotalus cerastes*; Sce = *Sistrurus catenatus edwardsii*; Ch = *Crotalus horridus*. See Table 1 for literature citations and test statistics.

Within this matrix, CWO (Fig 1) is constrained to montane woodlands between 1,475 m and 2,800 m elevation in three sky-islands (i.e., Animas Mountain = AMN, Peloncillos Mountains = PEL, Sierra San Luis Mountains = SSL) of southeastern Arizona, southwestern New Mexico, and north-central México (Fig 2). The species’ unique natural history, including extreme endemism, small population size, ancestral ecology, and acute over-specialization, not only promoted listing under the U.S. Endangered Species Act (ESA), but also illegal collection in the pet trade.

A complicating factor for its existence is the severity of wildfire that now predominates in ~30% of western U.S. forests, and integrates with low-severity fire in another ~45% [24], but with largest impacts in lower elevation dry-pine forests such as the sky islands [25]. Contemporary wildfire in western North America, as promoted by climate change, now extends over larger areas, exhibits higher intensity, and is more difficult to contain [26]. Extreme drought, high winds, and local topographies are exacerbating factors [27], as framed within a controversial program of fire suppression at federal and state levels [28, 29]. The combination of climate and disturbance is promoting a novel ecosystem in the sky islands, one with a pattern and process that are unlike those conditions that existed pre-disturbance.

### Demographic and Population Simulations

#### Demography and spatial ecology

Annual visual encounter surveys were conducted in late summer through early fall of each year from 1990 through 2008, and conducted both within and beyond the recognized distributions and habitats of the focal taxon. We estimated capture probabilities, population size and survivorship values [30] in the most data rich sky island population (i.e. ANM), using long-term (1990–2008) unpublished capture/recapture data based on 193 individuals tagged with passive integrated transponders [31, 32]. Demographic parameters (as above) were estimated by analyzing the 193 individual capture/recapture histories using the Cormack-Jolly-Seber [33, 34, 35] open population Maximum Likelihood (ML) estimator in the R-capture package [36] in R [37].

Adults (12 females, 15 males) were also radio-tracked (Model SB-2 transmitters, Holohill Systems Ltd) and located daily during 1994–1997 and 1999 (mean = 58±50 days; 1471 days total), using a Telonics radio-telemetry receiver (Model TR-4) and directional antenna [32]. Movements were recorded with a Trimble GPS Pathfinder system, or post-process corrected using Trimble GPS Pathfinder Office. Individual locations were converted from shapefiles to minimum convex polygons [38] with areas (in hectares) extracted, then tested for spatial differences among genders and life history stages using loglinear models in the R-capture package. These were compared against home range size of six North American rattlesnakes using analysis of covariance (ANCOVA), with gender and body size (snout-vent length, SVL) as covariates. Pairwise differences were examined for significance using Tukey’s Honest Significant Difference (HSD) test.

#### Population Viability Analysis

A prediction of population persistence is clearly an important aspect of risk assessment for SREs. Population viability analysis (PVA) incorporates systematic and stochastic impacts so as to calculate the fate of a population as well as its risk of extinction. It was applied to simulate the ANM population for 100 years across 10,000 iterations and 36 scenarios [39] with the following as input: reproductive maturity of females = age 4 (60% reproducing); males = age 2; both genders survive to age 11; mean number of offspring per brood = 5.4±1.6 individuals [40]. Population size = 151 individuals (derived from this study, as above), and survival rates were obtained via the ML approach outlined above.

#### Wildfire and Matrix Models

Prior to 1900, low intensity fires occurred on average every 6–7 years, as judged from fire scars on trees at Animas Mountain, whereas high intensity crown fires were less frequent [41]. We contrasted survival following low [42] and high intensity (2006) fires and employed three scenarios for context: (A) no fire, (B) low intensity fire, and (C) high intensity fire. Probability of each was determined using historical records [43], with survival estimated from mark-recapture data. Stable stage structure and reproductive values were also derived.

Sensitivity (numerical changes) and elasticity (proportional changes) are expedient results from which to predict impacts of small perturbations on vital rates of a population, with other elements held constant. These were subsequently employed to gauge impacts of wildfire on fecundity and survival. They are deemed influential when their summation is greater than one (sensitivity) or equal to it (elasticity).

### Genetic Impacts on Demography

While demographic parameters are essential for long-term persistence of populations, genetic factors are also important drivers of demography in small populations. We gauged contemporary population bottlenecks (i.e., <five generations; [44]) by contrasting heterozygosity estimates empirically derived from nine microsatellite (msat) DNA loci [15] against expectations under Hardy–Weinberg mutation-drift equilibrium (HWE). Sample size varied among sky islands: ANM (N = 54); SSL (N = 29); PEL (N = 18). Within ANM, samples were also subdivided into two areas: Indian Creek Canyon (AMN-IC, N = 24) and West Fork Canyon (AMN-WF, N = 30) [15].

A bottleneck is identified when the observed heterozygosity (*H*_O_) is significantly greater than expected (*H*_E_) for a population under mutation-drift equilibrium. Several statistical approaches are employed for adjudication, the most powerful being the Wilcoxon signed-rank test [45]. Microsatellite data were used to test for demographic independence among three sky island populations [15] and analyses were also conducted to evaluate the two ANM subpopulations (i.e., AMN-IC and AMN-WF; Fig 2)], using program BAYESASS 3 [46].

We simulated the change in the inbreeding coefficient (ΔF) for the ANM sky island population over 25, 50, 75 and 100-year spans, so as to evaluate if this parameter was potentially impacted by small population size. When ΔF is exceedingly large, individual fecundity will be markedly reduced and extinction risk concomitantly elevated, despite other mitigating processes [47]. However, ΔF calculations do not include gene flow and this aspect must also be evaluated as well, as done herein.

We also employed msat data to gauge the effective population size (*N*_e_) of each sky island population using two different ‘one-sample’ approaches: a linkage disequilibrium estimator (LDNe: [48]), and a Bayesian estimator (ONeSAMP: [49]). We also calculated the ratio of *N*_e_ to census population size (*N*_*c*_) for the ANM population and contrasted it with similar values taken from the literature and/or calculated for the European Adder (*Vipera berus*) [18]. We also estimated breeding population size (*N*_b_) using a two sex, no-sex-change model with a 15-year life span consisting of four non- and 11 reproductive years, with fecundity between two and nine [40], and survival empirically derived from mark-recapture analyses (as above). To account for the high variance of reproductive success, the Poisson factor was set at 2. We also generated *N*_b_/*N*_*c*_ and *N*_b_/*N*_e_ ratios, in that these are important from a management perspective. Generally, *N*_*c*_ can be estimated from ecological data whereas *N*_*e*_ requires genetic data not always (or rarely) available. However, *N*_*e*_ and *N*_*b*_ are more informative with regard to demographic and genetic processes that determine future population size.

### Modeling the Climate Envelope of an SRE

To predict the potential for habitat loss, 193 GPS capture coordinates were imported into MaxEnt [50] and parsed among training (N = 145) and testing (N = 48) sets. Nineteen bioclimatic variables were obtained from the WorldClim database [51] and a subset of eight biologically meaningful variables were selected due to correlations among variables [52]. Specifically, the climate envelope for CWO was derived using annual mean temperature, mean diurnal range, maximum temperature in the warmest period, minimum temperature of the coldest period, annual temperature range, mean temperature of the warmest quarter, mean temperature of the coldest quarter, and annual precipitation.

As CWO represents but one of five subspecies in the polytypic *Crotalus willardi* complex, and given the inherent complexity of modeling intra-specific entities [53, 54, 55], we excluded from calibration those areas where other *C*. *willardi* subspecies occurred. Thus, we do not implicitly assume absence where perhaps conspecifics with similar habitat requirements indeed exist. The extant niche for CWO was derived from 10,147 points, and averaged across 15 replicates at 5000 iterations each. A predictive species envelope was developed for the year 2080, based on a conservative climate projection of the Coupled Global Climate Model 2 (CGCM2) [1], and by averaging 15 replicates of 5000 iterations each. BioClim variables were assessed for relative contributions while information content was evaluated using the jackknife procedure. To ensure veracity of the projected climate envelope model (per [56, 57, 58]), a suite of standard regularization multipliers were tested (i.e., values of 1–10, 15, and 20). Improvement in model fit was determined using ENMTools [59, 60]. Mean distributional estimates for both models were imported into ArcGIS 10 to derive climate envelopes and core habitat areas.

### Ethics Statement

Collections were authorized via permits to Andrew T. Holycross by Arizona Game and Fish Department (HLYCR000038, SP605602, SP648632, SP711300, SP779370, SP841338); New Mexico Department of Game and Fish (2824); United States Fish and Wildlife Service (PRT676811, PRT814837). Specimens in México were collected under permit to J. Sigala-Rodríguez (DOO 750–3792/98). Animal care/handling was approved by Arizona State University (93–280R). The Animas Foundation, J. Austin, and C. Varela generously granted access to private lands.

## Results

### Demographic and Population Simulations

#### Demography and spatial ecology

**Fig 3:**
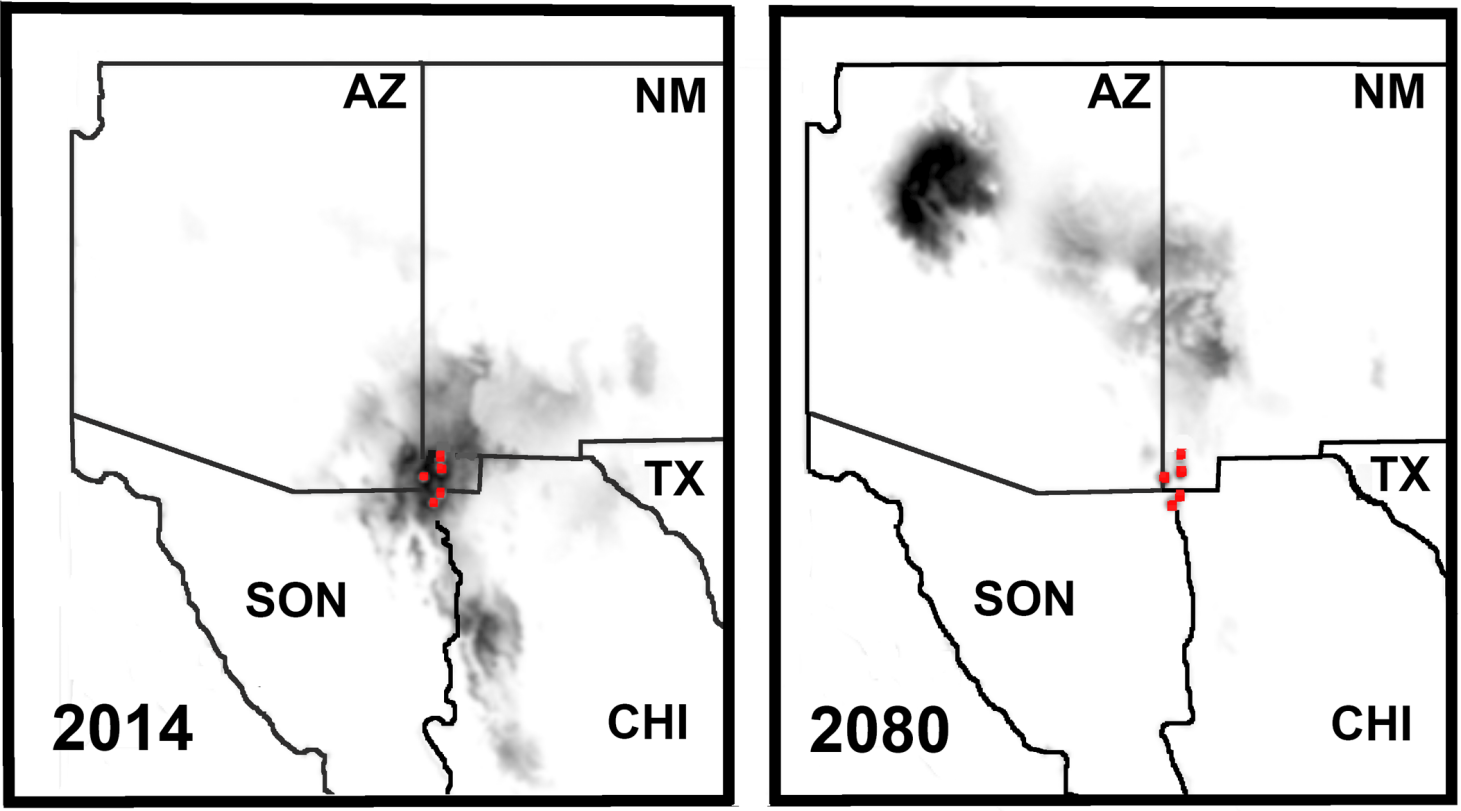
Bioclimatic variables (WorldClim database) incorporated with193 GPS capture coordinates for *Crotalus willardi obscurus* in the sky Islands of southwestern North America provide climate envelopes and core habitat areas in ArcGIS 10. Color density = strong habitat preference, with black/dark grey being most positive. Red circles = sampling locations. 2014 = current climate envelope; 2080 = a conservative climate envelope projected 66 years in the future.

A total of 96 adults and 97 juveniles were captured over 18 years. Adult sex ratio was = 1:1. Survival rate was significantly higher for males, but did not differ between females and juveniles. Capture probabilities were 0.21±0.06 for adults and 0.39±0.12 for juveniles, respectively (Table 1). Adult home ranges were small (mean = 0.07±0.2 km^2^), overlapping, and not significantly different by sex, body size, or year, with non-significant interactions. Yet, home range estimate for CWO differs significantly from those recorded for six rattlesnake species (Table 2; Fig 3), irrespective of gender or body size.

**Table 1:** Population demographic parameters for *Crotalus willardi obscurus* (= CWO) in the Animas Mountain sky island compiled by life history stage and population.

Comparison	df	Survival	Capture	Pop. Size
Male vs Female	141	6.65**	5.81**	7.40**
Male vs Juvenile	121	11.46**	4.66**	1.91**
Female vs Juvenile	63	0.041	0.044	1.31
Adult vs Juvenile	190	13.77**	4.91**	0.78

Comparison = Life history stages evaluated; df = degrees of freedom; Survival = F-value for comparison of survival by life history stage; Capture = F-value for comparison of capture probability by life history stage; Pop. Size = F-value for comparison of population size by life history stage.

** = Statistical significance at P < 0.05.

**Table 2:** A Test of home range estimates for *Crotalus willardi obscurus* (= CWO) and six other rattlesnake species for which data are available, using analysis of covariance (ANCOVA) with gender and body size as covariates.

Taxon	N	Home Range	P	Data Source
*Crotalus adamanteus*	10	31.5 ±23.40	0.001	[77]
*C*. *cerastes*	25	23.2 ±13.99	0.013	[76]
*C*. *horridus*	20	25.7 ±11.3	0.006	[80]
*C*. *oreganus abyssus*	7	11.3 ±10.74	0.014	[106]
*C*. *willardi obscurus*	27	7.1 ±19.76	N/A	[This study]
*Sistrurus c*. *catenatus*	11	25.3 ±24.89	0.007	[78]
*Sistrurus c*. *edwardsii*	12	42.0 ±29.42	0.0001	[79]

Significant post-hoc pairwise differences are identified using Tukey’s Honest Significant Difference (HSD). Taxon = genus/species, where *C*. = *Crotalus* and *c*. = *catenatus*; N = number of individuals per study; Home Range = hectares with variance; P = Statistical probability of a larger value; Data Source = Literature citation.

#### Population Viability Analysis

All 36 PVAs for the ANM sky island yielded markedly negative mean population growth (r = -0.20±0.03) that continued to decrease as environmental and demographic stochasticity were incorporated (r = -0.22±0.03). Probability of extinction was 100% with mean time to extinction (TtE) = 17.09±2.05 years. Adults decreased in prevalence as body size increased, with larger snakes having greatest reproductive value (59.5%) yet comprising only 3.6% of the population. Effects of inbreeding on population growth are severe, irrespective of deterministic or stochastic models (Table 3). Estimates of TtE also decreased sharply as inbreeding depression increased.

**Table 3:** Declines in population growth (= r) for *Crotalus willardi obscurus* in the Animas Mountain sky island under four levels of inbreeding depression (i.e., None; Low; Moderate; High), as gauged using both deterministic and stochastic population models.

Inbreeding Depression	r (deterministic)	r (stochastic)	TtE
None	-0.167	-0.185 (± 0.329)	19.6
Low	-0.192	-0.206 (± 0.325)	17.8
Moderate	-0.244	-0.244 (± 0.335)	14.7
High	-0.29	-0.311 (±0.33)	11.3

TtE = Time to extinction, in years.

#### Wildfire and Matrix Models

Adult and juvenile survivorship was 62% and 46%, respectively, and declined significantly in years with wildfire. For the prescribed, low intensity fire of 1997, survivorship for adults decreased 13% vs 7% for juveniles, and 20% vs 23% during the high intensity fire in 2006. Sensitivities and elasticities were relatively uniform for ‘no fire,’ low intensity fire,’ and ‘high intensity fire’ categories (Total, Table 4), with adult survival more seriously impacted. However, a disproportionally larger increase was found for juveniles regarding sensitivity to high intensity fire.

**Table 4:** Sensitivity and elasticity estimates as derived from a population viability analysis (PVA) for *Crotalus willardi obscurus* (CWO) in the Animas Mountain sky island (New Mexico, U.S.A.).

			Fire Regimes	
		No Fire	Low Intensity	High Intensity
**Sensitivity**	Fecundity	0.27	0.29	0.21
	Juvenile Survival	0.53	0.55	0.71
	Adult Survival	0.86	0.83	0.85
	TOTAL	1.66	1.67	1.77
**Elasticity**	Fecundity	0.23	0.26	0.24
	Juvenile Survival	0.23	0.26	0.24
	Adult Survival	0.54	0.47	0.53
	TOTAL	1	1	1

Fecundity and juvenile and adult survival are estimated over 100 years under three Fire Regimes (= No Fire; Low Intensity Fire; High Intensity Fire).

### Genetic Impacts on Demography

Standardized and Wilcoxon-Ranked tests detected significant and contemporary bottlenecks in all sky island populations. Additionally, ΔF over 25, 50, 75 and 100 years increased steadily from 0.31 (25 years) to 0.52, 0.66 and 0.77, respectively. Estimates of *N*_e_ using the LDNe model were 65, 29, and 39 for ANM, PEL, and SSL sky islands, respectively, with similar values (i.e., 70, 25, and 42) produced by the ONeSAMP procedure. The latter also yielded estimates for Animas subpopulations: AMN-IC (= 36) and AMN-WF (= 43). The estimate for number of breeding adults (*N*_b_) in ANM is 24, while that for the Adder in Sweden is 17. Ratios of *N*_b_/*N*, *N*_e_/*N*, and *N*_b_/*N*_e_ for CWO fall below (two comparisons) or match (one comparison) those for the Loggerhead Sea Turtle, whereas they match (one) or exceed (two values) for the Adder (Table 5).

**Table 5:** Estimates for census size (= N), number of breeders (= Nb), and effective population size (= Ne) for the New Mexico Ridge-nosed Rattlesnake, *Crotalus willardi obscurus* (= CWO) in the Animas Mountain sky island.

Parameter	CWO	CC	VB
N	151	n/a	166
N_b_	24	n/a	17
N_e_	38	n/a	28
N_b_/N	0.159	0.188	0.102
N_e_/N	0.252	0.231	0.169
N_b_/N_e_	0.632	0.811	0.607

Metrics for Loggerhead Sea Turtle, *Caretta caretta* (= CC) from literature, and the Adder, *Vipera berus* (= VB) from literature or estimated in this study. Ratios of the various parameters with census size (N) are also provided.

Mean migration rate was calculated as 2% among all sky island populations, substantiating their demographic independence [61]. However, migration rate between the two ANM subpopulations was surprisingly unidirectional, with those from Indian Creek (ANM-IC) to West Fork Canyon (ANM-WF) at 75.0%, whereas movements from ANM-WF to ANM-IC were but 23.0%. These data suggest a source-sink dynamic in the ANM sky island, a situation similar to that found for populations of an endangered venomous snake in Australia [62].

### A Shifting Climate Envelope for an SRE

The climate envelope for CWO displayed an Area Under the Receiver Operating Characteristic (AUC) value of 0.997 (±0.001), indicating an evaluation that was well supported and strongly predictive. Mean diurnal range emerged as the most important variable (43% contribution), whereas mean-temperature-of-coldest-quarter, minimum temperature of coldest month, and annual precipitation were also strongly predictive (29.8%, 20.6, and 5.4% contribution, respectively). Model selection that varied the regularization parameter (beta) among12 models revealed that the best model (AICc = 1792.23, ΔAICc = 0.00) was that with beta value of 6. The next best (AICc = 1802.23, ΔAICc = 10.00) had a beta value of 7. Thus, climate envelope maps were derived from the average outputs of the beta = 6 regularization parameter models.

**Fig 4:**
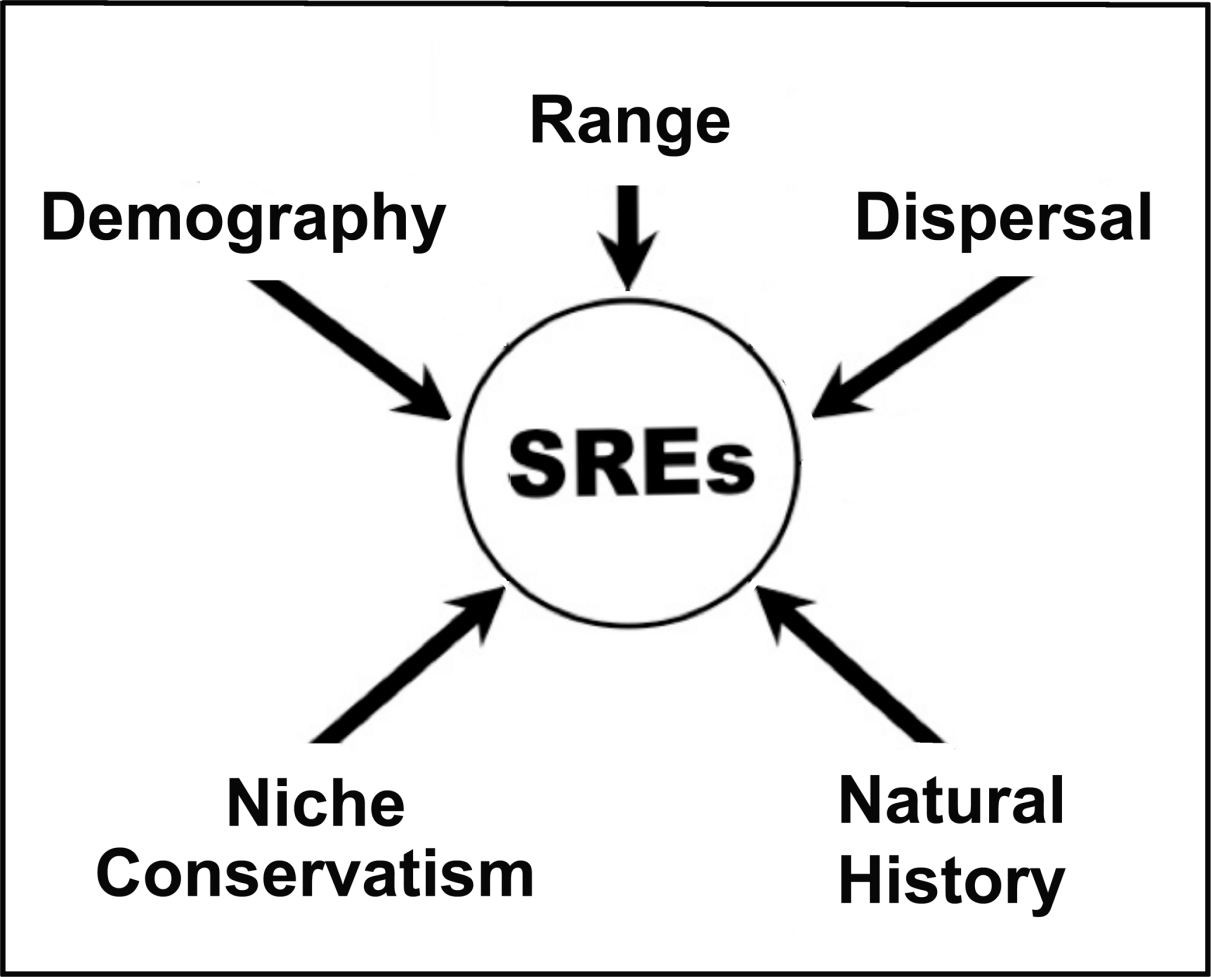
A conceptual diagram illustrating five key life history components that predispose short range endemics (= SREs) to extinction. All five must be assesse to appropriately gauge long-term persistence of SREs in imperiled ecosystems.

CWO has a relatively restricted climate envelope with predicted core areas juxtaposing well with designated critical habitat (Fig 4). However, a scenario of moderate climate change over some 67 years (i.e., to 2080) would shift the current distribution to the extreme periphery of the climate envelope, with the core area migrating approximately 763km north to the San Francisco Peaks (on the Colorado Plateau, near Flagstaff, Arizona), a geographic extension considerably beyond the historic range of CWO.

## Discussion

### Demographic Processes, Movement, and Comparative Studies

Reducing loss of biodiversity is a conservation challenge entailing fundamental biological as well as applied management dimensions, and is constrained by finite resources and sparse data. The problem is even more austere when its foci are small populations and SREs. One potential solution, and a mechanism to more fully understand extinction vortices, is to approximate population trends through the application of demographic models [63]. Here, four sources of uncertainty are recognized: Low quality data; difficulties with parameter estimation; issues with validation; and limited modeling alternatives [64]. The first two are particularly germane to this study, as few CWO remain in but three isolated sky islands. Yet model uncertainty also hinges on the spatio-temporal scope of sampling, and can be mitigated by a careful design [65]. In the present study, two different research teams extensively sampled all known CWO populations over the span of two decades. Additionally, demographic parameters were estimated via maximum likelihood (ML), an approach that minimizes sampling error in lieu of process error [66]. Finally, multiple models were employed, with those individually based having greatest utility in capturing small population fluctuations [67], and those results are reported herein.

Long-term studies similar to ours in both taxonomy and scope reveal population dynamics that are quite congruent with those found herein. A 10-year mark-recapture study on the federally threatened Eastern Indigo Snake (*Drymarchon couperi*) also found negative population growth coupled with low annual survival and reproduction [68]. Likewise, a 20-year demographic study on the Mediterranean Dice Snake (*Natrix tesselata*) enumerated low mean annual survival (r = 0.73; [69]) that is comparable both to the above study as well as ours. Given these results, we hypothesize that a survival rate approaching 0.70 for snakes, when combined with relatively low fecundity, is a metric that can be utilized to validate a declining population.

Developing generalized models of snake movement is difficult [70, 71, 72] due to myriad intrinsic [73, 74, 75] and extrinsic [76, 77, 78, 79] factors and their manifold effects [80]. Yet comparisons among taxa can be particularly valuable in assessing spatial pressures exerted upon species particularly prone to extinction (e.g. endangered species, SREs, etc.) versus those less restricted forms [81].

A qualitative comparison between the New Mexico Ridge Nosed Rattlesnake and the Twin Spotted Rattlesnake (*Crotalus pricei*, home range = 0.031 ± 0.009 km^2^; [82], an Arizona state protected Sky Island SRE allopatric with the New Mexico Ridge Nosed Rattlesnake, reveals similarly diminuntive home range sizes and restricted movements. This suggests that Sky Island rattlesnake SREs may share similar restrictions upon movement that preclude them from successful long-range dispersal, or range expansion. Direct quantitative comparisons with non-SRE North American rattlesnakes exhibiting substantially greater geographic distributions reveals the New Mexico Ridge Nosed Rattlesnake displays significantly reduced movement patterns.

Ultimately, these comparisons reveal that the New Mexico Ridge Nosed Rattlesnake is of low vagility and with restricted dispersal capability. This places the form in a precarious position, given predictions of a substantially shifting climate envelope depicted herein. Individuals should continue to track conditions that shift into higher elevations on their respective mountains, yet this represents an increasingly small area of suitable habitat. Therefore, this SRE will likely sink into an extinction abyss as climate change and vicariant desertification proceed unabated up these mountains.

### Genetics and Demography

Tests for heterozygosity-excess are quite conservative and can often fail to detect the signal of a bottleneck, despite recognized and historic declines [44]. However, our tests revealed strong support for recent and severe population reductions across the sky islands, and sustain an argument that the small population paradigm [83] is preemptive in driving the extinction vortex for our SRE. Additionally, ΔF-calculations reveal inbreeding values that elevate substantially as temporal span lengthens, underscoring the considerable impact of inbreeding as it relates to fitness over time.

Effective population size (*N*_e_) is another parameter that strongly impacts threatened and endangered populations, more so than selection, immigration and emigration [84]. Here, *N*_e_ estimates are gauged against a hypothetical population with constant population size, equal sex ratio, and with drift or inbreeding comparable to the study population, but without immigration, emigration, mutation, or selection. Conservative estimates for closed populations (as herein) are represented by the ‘50/500 rule’ (i.e., Franklin’s Rule), with 50 as a basis for short-term survival with minimal inbreeding, and 500 for long-term survival. This metric has been extant for 3.5 decades and is now deemed inadequate, with more contemporary values being >100 (lower bound) and >1000 (upper bound) [47]. Regardless, our *N*_e_ estimates were below the legacy lower bound in all sky islands and consequently elicit concern as they imply an immediate extinction risk. Similarly, *N*_b_ (the effective number of breeders per year, a value generally lower than *N*_e_) is likewise diminished and thus elicits managerial apprehension for the ANM population.

Extrapolations of *N*_e_ with *N* as a measure of genetic drift have likewise evolved, and management actions that can elevate *N*_e_ or the *N*_e_ **/** *N* ratio (and thus genetic variation) have grown in importance. An earlier consensus that argued for a ratio averaging 0.10–0.14 has subsequently been expanded to 0.20, but with the caveat that it should be juxtaposed with the life history of the species in question [47]. In this sense, *N*_e_/*N* can be arbitrarily large for species with prolonged juvenile and short adult lifespan, such as insects.

Ratios of *N*_e_/*N* effectively link demographic and evolutionary processes across a wide range of taxa, as reflected by the fact that half its variance is explained by only two life-history traits (i.e., age-at-maturity and adult lifespan). The value for the ANM population (i.e., 0.252) is lower than that recorded in five (of six) reptiles for which data have been recorded (Table S-2 in [84]). It approximates that for Loggerhead Sea Turtle [an endangered species under the ESA and on the Red List of the International Union for Conservation of Nature (IUCN), but exceeds the value recorded for the European Adder in Sweden (Table 3). The *N*_b_/*N* value for ANM (= 0.159) is also lower when compared with the six listed reptilian species [64], to include the Loggerhead Sea Turtle (at 0.188), but again exceeds somewhat the value for the Adder. Lastly, the *N*_b_/*N*_e_ ratio (= 0.632) is again lower than those for the six listed reptiles [84], to include the Loggerhead Sea Turtle at 0.811, and also comparable to that of the Adder (at 0.607). Overall, the same two life history traits (as above) explain 67% of the variance in *N*_b_/*N*_e_.

The comparisons we draw between CWO and the European Adder in southern Sweden (above) are particularly germane, in that the Adder is a recognized conservation icon that epitomizes the impacts of reduced genetic variability on population demography (see texts by [85, 86, 87]). It was originally recognized [18] as a severely inbred and isolated population, and 20 males were translocated from a more distant population in an attempt at genetic rescue. This significantly enhanced the genetic variability of the study population and prompted a dramatic increase in offspring viability as well as a rapid growth in numbers. It had the effect, at least in the short term, of halting the decline of the population towards extinction. Unfortunately, continued fragmentation has again condensed the population [21], and its recognition as an exemplar in the conservation literature could not stem the increasing urbanization of its habitat.

The situation in the sky islands is a reflection of that with the European Adder in southern Sweden, but without the attempt at genetic rescue (but see below). The comparative relationships among the various demographic ratios, as presented above, underscore the tenuous genetic and demographic status of our Madrean SRE and, much like the Adder in southern Sweden, offer scant promise for longevity. The demographic trajectory of CWO in the sky islands will inexorably lead to extinction unless an adaptive management plan is rapidly initiated.

### Does Wildfire Significantly Influence Survival at Animas Mountain?

A policy of fire suppression in western North America, enacted by the U.S. Forest Service in late 19^th^ century [41], has largely eliminated wildfire as a natural process, consequently provoking drastic alterations in structure, composition, and fuel load of western North American woodlands [43]. Wildfire promotes vicariant desertification at lower levels in the sky islands, yet the capability for movement to suitable habitat at higher elevation is curtailed in that topographically there is no ‘up’ remaining. From a latitudinal perspective, a predicted shift in the climate envelope >700 km to the northwest (Fig 4) is also insurmountable, not only for persistence of CWO but the entire Madrean Archipelago. Clearly, wildfire has a strong effect on survivorship at all life history stages, with fires of high intensity significantly impacting juveniles (Table 4).

Yet, catastrophic fire is not a predominant component in the extinction rate for CWO (Table 4). Point estimates gathered from low and high intensity wildfire (i.e., 1997, 2006) are indeed statistically significant, yet infrequent and of little consequence over longer temporal spans, but with one important caveat: for this to ring true, the core structural elements of the Madrean Pine-Oak ecosystem must remain intact. Within such an historic system, small population processes are instead more manifest for long-term survival [88]. Furthermore, our data suggest that once a population is propelled by demography into an extinction vortex [89], its dice are effectively cast and the decline cannot be countered, only promoted. Here, one such influential propellant is rapid climate change [90] that, in turn, elicits wildfire as an instantaneous response [91]. This is indeed an unfortunate juxtaposition in that the role of fire in the Madrean ecosystem is currently transitioning from an historic ‘rejuvenator’ of the ecosystem to one more contemporary and abrupt, i.e., that of ecosystem ‘converter.’ Its end points are a new species composition and a strong resistance to historic relapse, both cued by climate change with severe wildfire as its handmaiden [92]. These aspects are immensely important for both CWO and the Madrean Archipelago, and will have clear impacts in the near term.

### Are SREs Predisposed to Extinction?

One argument to the affirmative is the recognition that distributions of SREs are relictual, restricted, and easily perturbed by anthropogenic fragmentation and stochastic events (e.g. wildfire, flood, drought most often associated with climate change) ([17]; see above, but also [93]). These events easily dissociate SREs from their contemporary climate envelopes (per Fig 4) and promote strong, negative selection on those elements more specialized in their life histories [94]. Thus an understanding of the life history characteristics that define SREs, and which predispose them for extinction, not only elevate concerns (as herein), but can also promote management scenarios that may enhance long-term persistence. With regard to CWO, the promotion—even reintroduction—of fire within a dramatically altered Madrean Pine-Oak ecosystem can indeed be such a selective pressure that accelerates decline.

### Implications for Conservation

One approach to alleviate the adverse effects of inbreeding and genetic isolation in CWO would be to re-establish gene flow among impacted sky island populations (as was done with the European Adder in southern Sweden). Yet, in spite of potential success, there have been only 19 global instances where this has been implemented in a threatened and near-threatened species [95]. One obvious concern is the potential for outbreeding depression (OD), defined as a reduction (rather than augmentation) of reproductive fitness during the first (or subsequent generations) post-supplementation, and stemming from an admixture of ill-adapted genotype complexes. The occurrence of OD has been documented in some 35 species [96], with risks, particularly in the second generation, recorded as on par with those of inbreeding. However, others have argued that the topic is not only overemphasized in the literature, but also overstated by conservation managers as an element of concern [95].

The re-establishment of natural or artificial gene flow via introductions now has a contemporary designation (i.e., ‘genetic rescue’) although this interpretation has become further dissected in the literature [97]. Many managers view the approach as positive, due largely to its acknowledged success in relatively dire situations: Florida Panther [98], Bighorn Sheep [99], Greater Prairie Chicken [100], as well as the aforementioned European Adder. It has also been substantiated experimentally in the laboratory, where crosses among severely bottlenecked strains of *Drosophila* reversed the effects of inbreeding and promoted reproductive success. These conditions persisted into the second generation of hybrid offspring, whereas those crosses within (rather than across) strains showed little benefit [101].

However, genetic rescue does have limitations, and these pertain specifically to the present study. For example, results were limited when supplementation for genetic rescue has occurred, largely due to a severely degraded habitat such that isolation and subsequent inbreeding were instead promoted [102]. Indeed, this is the situation within the sky islands, where fragmentation has been furthered both by desertification at lower elevations and wildfire at higher elevation. Each process has curtailed available habitat and, in so doing, promoted the onset of small population effects, a situation analogous to the European Adder in southern Sweden.

Biodiversity hotspots clearly sustain SREs, and their management is a global mandate. Yet a serious conservation challenge is the manner by which habitats and species can be not only protected but also augmented. CWO underscores the seriousness of this issue. It is listed as ‘threatened’ by the U.S. Fish and Wildlife Service [103] and indeed this confers protection. It is the only venomous reptile (and one of but 12 snakes) that has received such designation. Yet despite ~40 years of ‘protection,’ its evolutionary trajectory continues to diminish. Does this represent an inability of the ESA to indeed protect listed species? And if so, is such a conclusion realistic?

We suggest the argument should instead be posited as: “How can this ecosystem and its unique biodiversity be more appropriately conserved?” Here, we offer three recommendations: First, CWO should be immediately elevated to ‘endangered’ status, as this will leverage increased ecosystem management for the sky islands in their entirety [104]. Second, CWO should be promulgated as an exemplar of climate change impacts, and thus as a component of risk analysis under the ESA [105]. Finally, other uniquely endemic SREs in the sky islands should also be identified as flagship species, so as to promote public awareness as well as shape stakeholder perceptions regarding ecosystem conservation. We recognize these actions may not save but merely prolong its existence, but this in itself would be positive in that it would buy time so that more substantive ecosystem-level initiatives can be developed in the context of region-specific mandates [92, 2].
